# Natural wind variability triggered drop in German redispatch volume and costs from 2015 to 2016

**DOI:** 10.1371/journal.pone.0190707

**Published:** 2018-01-12

**Authors:** Jan Wohland, Mark Reyers, Carolin Märker, Dirk Witthaut

**Affiliations:** 1 Institute for Energy and Climate Research (IEK-STE), Forschungszentrum Jülich, Jülich, Germany; 2 Institute for Theoretical Physics, University of Cologne, Cologne, Germany; 3 Institute for Geophysics and Meteorology, University of Cologne, Cologne, Germany; University of Liverpool, UNITED KINGDOM

## Abstract

Avoiding dangerous climate change necessitates the decarbonization of electricity systems within the next few decades. In Germany, this decarbonization is based on an increased exploitation of variable renewable electricity sources such as wind and solar power. While system security has remained constantly high, the integration of renewables causes additional costs. In 2015, the costs of grid management saw an all time high of about € 1 billion. Despite the addition of renewable capacity, these costs dropped substantially in 2016. We thus investigate the effect of natural climate variability on grid management costs in this study. We show that the decline is triggered by natural wind variability focusing on redispatch as a main cost driver. In particular, we find that 2016 was a weak year in terms of wind generation averages and the occurrence of westerly circulation weather types. Moreover, we show that a simple model based on the wind generation time series is skillful in detecting redispatch events on timescales of weeks and beyond. As a consequence, alterations in annual redispatch costs in the order of hundreds of millions of euros need to be understood and communicated as a normal feature of the current system due to natural wind variability.

## Introduction

In the last years renewable generation capacity has grown strongly while costs have decreased substantially [1]. Since 1990, electricity generation in the OECD from wind has increased by a factor of 158 (to 600 TWh in 2016) and photovoltaic generation has increased by a factor of 11500 (to 218 TWh in 2016) [2]. The energy portfolio has thus changed substantially since the first publication of evidence for anthropogenic climate change by the Intergovernmental Panel of Climate Change [3].

In Germany, for example, the relative contribution of renewables to overall electricity generation reached roughly 33% in 2016 [4] and it is planned to increase further. Installed capacity in the German wind sector alone totalled roughly 50 GW in 2016. To give an impression of scale, this implies that electricity demand could theoretically be balanced by wind generation on a windy Saturday. Since consumption is higher during the week, current installed wind capacities are not yet sufficient to cover weekday consumption entirely. However, this will likely be the case in a few years time. Onshore wind energy is widely considered a least-cost energy source and photovoltaic (PV) cells are about to enter this domain as well [5]. Additionally, in April 2017, a major German offshore wind park won acceptance of its bid without any subsidy at all which indicates the economic viability of the technology [6]. The relative attractiveness of wind and solar power plants in contrast to conventional power plants would still increase if large pre-tax subsidies for coal were included in the economic assessment [7].

This development is promising in the sense that fast decarbonization of the electricity system is economically feasible. A carbon-neutral electricity system is a fundamental ingredient in restricting climate change in line with the Paris Agreement which aims to avoid dangerous interference with the climate system [8, 9]. In order to reach the ambitious Paris goals, however, decarbonization needs to be accelerated and extended to sectors other than electricity [10–12]. Recent research has revealed that sector coupling and the usage of flexible loads allows the creation of functional and cost-efficient energy systems fueled by renewables only [13–16].

**Fig 1:**
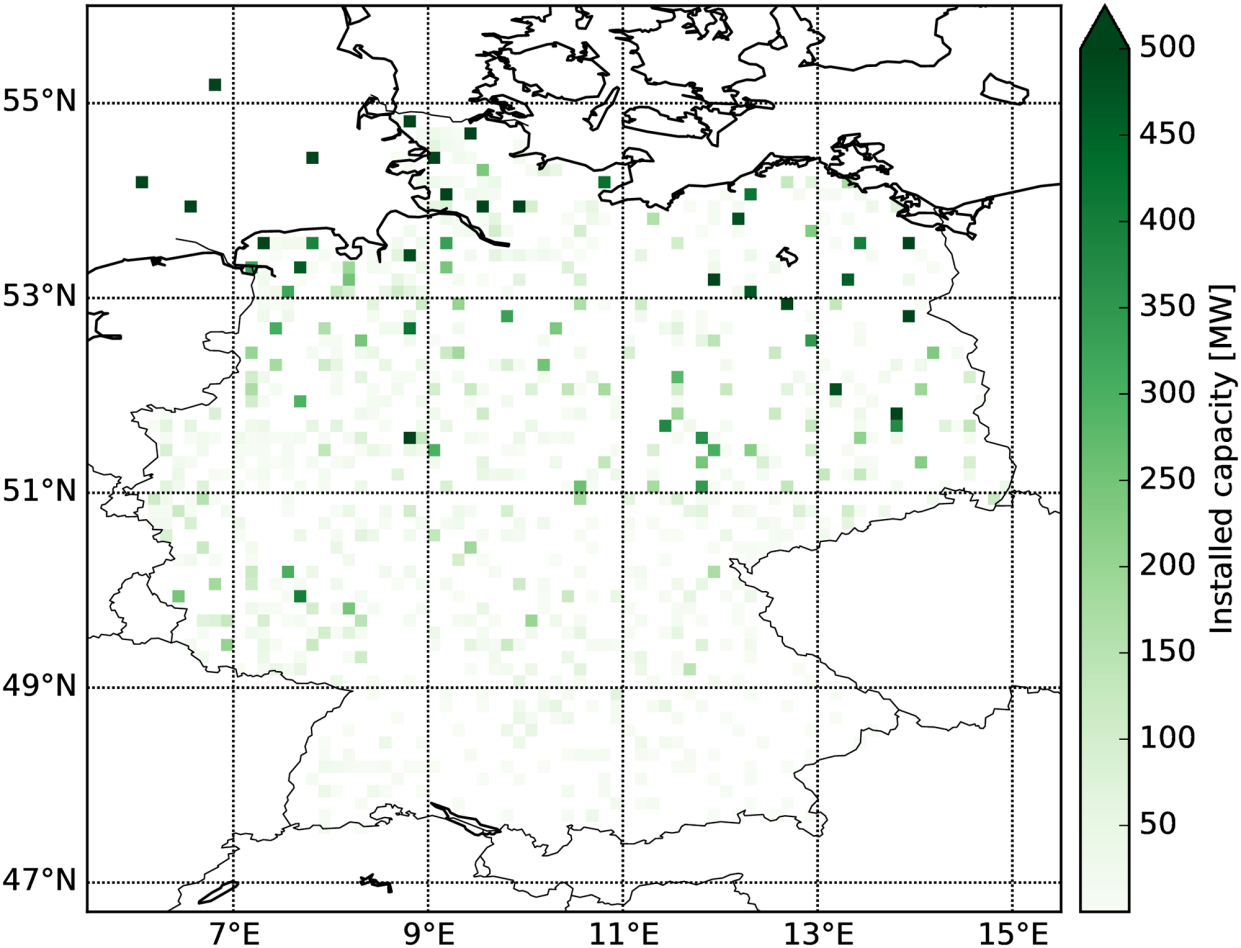
Allocation of wind parks used in this study. Note that an upper bound of 500 MW is set for the colorbar to ensure visibility of smaller parks. The biggest installed capacity per grid cell is around 1.4 GW. Wind park data is taken from the OPSD database for end of 2016 [53]. Offshore wind parks are not georeferenced in the input data and they are equally distributed to the four biggest operational offshore wind parks (Bard, Borkum Riffgrund, Amrumbank West, Sandbank).

However, non-dispatchable and intermittent renewable electricity generation poses a challenge for grid integration. This challenge expands as renewable penetration increases and regulatory means to ensure system stability are needed (e.g., [17]). In Germany, renewable generation is given priority for grid feed-in. As the centers of, in particular, wind generation and electricity demand are spatially separated, large amounts of electricity need to be transmitted across the country. The north-south gradient of wind park allocations is presented in Fig 1. Since the transmission system has not been initially designed to serve this purpose, overloading and congestion in times of high renewable generation occurs (e.g., [18]). Adaption of the grid via new or enlarged transmission lines is planned, yet involves timescales of multiple years to decades.

According to the German Energy Act (‘Energiewirtschaftsgesetz’), Transmission System Operators (TSOs) and Distribution System Operators (DSOs) are in charge of maintaining energy system stability at all times [19]. In order to achieve this, four strategies can be applied: redispatch (shifting conventional generation in space by increasing and decreasing generation of conventional power plants in comparison to the initial market-based dispatch), usage of reserve plants (ramping up conventional power plants from a specific pool of plants, ‘Netzreserve’), feed-in management (reducing renewable generation, ‘Einspeisemanagement’) and lastly adaption measures (emergency measure to reduce generation, ‘Anpassungsmassnahmen’). These measures increase overall system costs because plant operators are compensated for having to reduce generation which is in addition to the costs of increasing generation elsewhere. As an exception, the adaption measures are not paid for since they are used in emergency cases only.

Germany saw a large increase in redispatch and feed-in management in 2015. Redispatch (sum of reductions and increases) was used to control about 15.4 TWh of electricity at a total cost of € 412 million, reflecting a threefold increase as compared to the previous year. Both the usage of reserve plants and feed-in management increased substantially as well [20]. The federal network agency for electricity, gas, telecommunications, post and railway (Bundesnetzagentur) lists a couple of reasons for these sharp increases: Besides the strong increase in wind capacity on land, the commissioning of two conventional power plants in the north and decommissioning of one nuclear power plant in the south added to the spatial mismatch of generation and load. This mismatch was exacerbated by substantial electricity exports to Austria. Moreover, grid extensions required temporal shutdown of grid elements and grid expansion in general was lagging behind schedule. As an aside, there is disagreement as to whether the grid is currently limiting the progress of the energy transition or whether it just sets a limit to exporting electricity from cheap coal-fired plants [21].

The issue of escalating grid management costs, including redispatch, even entered the public debate, particularly in late 2015 and January 2016, as numerous newspaper articles show (e.g., [22–25]). Since redispatch costs contribute to overall grid fees which accounted for around one fifth of the electricity price in 2016, they directly influence the energy costs of consumers [26]. This might have influenced the timing and content of modifications made to the Renewable Energy Act (“Erneuerbare Energien Gesetz”, EEG) in July 2016 [27]. The main goals of the modifications are to reduce subsidies via competition between investors and to provide a steering mechanism which controls the siting of new projects and also limits installed capacities. In particular, it includes a restriction for onshore wind parks in regions with a high probability of congestion while system-friendly installations are incentivized [27]. In other words, the allocation of new parks is regulated in order to reduce future increases of grid management costs. This part of the EEG reform can be seen as an attempt to reduce redispatch costs by synchronizing the expansion of grids and renewable energy sources. Since the reform only entered into force in early 2017, it cannot have influenced the 2016 figures.

Unexpectedly, redispatch and feed-in management substantially decreased in 2016. The redispatch volume dropped to 11.5 TWh (25% reduction) at a cost of € 220 million (46% reduction). Although there was a small increase in reserve plant usage from 0.6 TWh to 1.2 TWh at the same time, it cannot explain the stronger change in redispatch because of its small magnitude.

What caused the substantial drop in redispatch costs? What does a likely evolution for the years ahead look like? In this paper, we address these questions based on the hypothesis that natural interannual variations of the wind resource caused the drop. There is a multitude of other potential reasons for redispatch. For example, other volatile types of renewable generation, namely solar photovoltaics, could lead to redispatch during times of high generation. Since the German electricity grid is currently being expanded in order to meet the changing needs, temporal shutdown of grid elements during this expansion might also contribute to redispatch. Moreover, electricity exports to neighboring countries can increase the loads and thereby exacerbate congestion. Scheduled downtimes of conventional plants for maintenance might have also played a role. All of these other reasons do not depend directly on the wind resource and are hence not dominant if our hypothesis can be validated. In contrast, if natural variability dominates the redispatch variability, it also has a strong impact on the technological and economic aspects of the energy transition. It would follow that more attention should be paid to assessing and dealing with climate-induced uncertainty. Therefore, the interplay of energy and climate in general would need further investigation. This is especially true since installed wind capacities will continue to increase.

## Background

Wind fluctuates naturally on timescales from seconds to multiple years and so does wind power generation (e.g., [28–32]). In addition to understanding the variations themselves, it is important to quantify their impact on the power system and the associated costs. This is partly because the costs for renewable power generation are intrinsically linked to system costs [33, 34]. Focusing on the US, [34] found that installation and maintenance costs are not sufficient to characterize the actual costs of renewables if the renewable gross share exceeds around 30%. Instead, system costs from balancing mismatches between volatile generation and load need to be incorporated. Interestingly, 2015 was the first year during which renewables contributed more than 30% to German electricity production.

In principle, there are well-known options to reduce the vulnerability of the power system to wind variability. For example, wind fluctuations can be compensated by PV fluctuations thus smoothing the renewable generation time series [35]. Storage and intercountry balancing facilitate system stability [36–38]. Moreover, a multinational optimization of future wind park allocations would allow for a substantial reduction of volatility [39]. This effect is amplified under strong climate change due to changes in wind correlations [40]. In this context, it is also important to study the co-evolution of renewable generation and electricity consumption. In places where a substantial fraction of heating (or cooling) is provided by electricity, a strong annual cycle of electricity consumption is expected. [41] found that wind generation generally decreases synchronously with increasing consumption in winter in Great Britain, which implies that wind power is not well suited to cover winter demands there. However, as an exception to this tendency, they also report that the wind power generation partly recovers at the highest consumption events. Moreover, [42] identified a spatial shift in electricity consumption as a consequence of climate change. Its amplitude increases with the level of greenhouse gas concentrations in the atmosphere. If carbon emissions continue to rise in the future, this effect will thus have to be accounted for in long-term energy system planning.

We expect wind generation to trigger redispatch events because it features a substantial spatial mismatch between generation and consumption in Germany. This is in contrast to PV, which is strongly deployed in the south and thus closer to major industries [18]. Moreover, the diurnal cycle of solar generation resembles the daily load profile in principle and is thus rather system friendly (at current levels of installed capacities). Wind power generation also varies stronger with wind speeds (cubic dependency within a certain range) than PV generation varies with incoming irradiance (linear dependency) [43]. We thus investigate the interrelation between redispatch and wind generation here.

## Methods and data

Generally speaking, we used high-resolution weather data to calculate wind generation and investigate its relationship to the redispatch time series of 2015-2016. High-resolution weather data, in contrast to ex post generation data, has the advantage that long time series of multiple decades exist and thus natural climatic variability can be accounted for. Moreover, it isolates the impact of weather, which is masked by increases in capacity in ex post data.

### Wind generation based on ERAINT

More precisely, we calculate wind generation *G*_Wind_(*t*) based on the ERAINT reanalysis on 0.11° angular resolution (roughly 12km) and 6 hour time steps [44]. ERAINT has a native grid spacing of 0.75° and the increase in resolution to 0.11° is achieved via bilinear interpolation done by the climate modelers. The dataset is available from 1979 and is regularly updated. In particular, the years 2015 and 2016 are included. Reanalysis data combines the advantages of model results and measurements in the sense that it (a) gives data on regular grids which (b) is also based on observations. It is for this reason that reanalyses have already been widely used for energy-related assessments [45–52].

**Fig 2:**
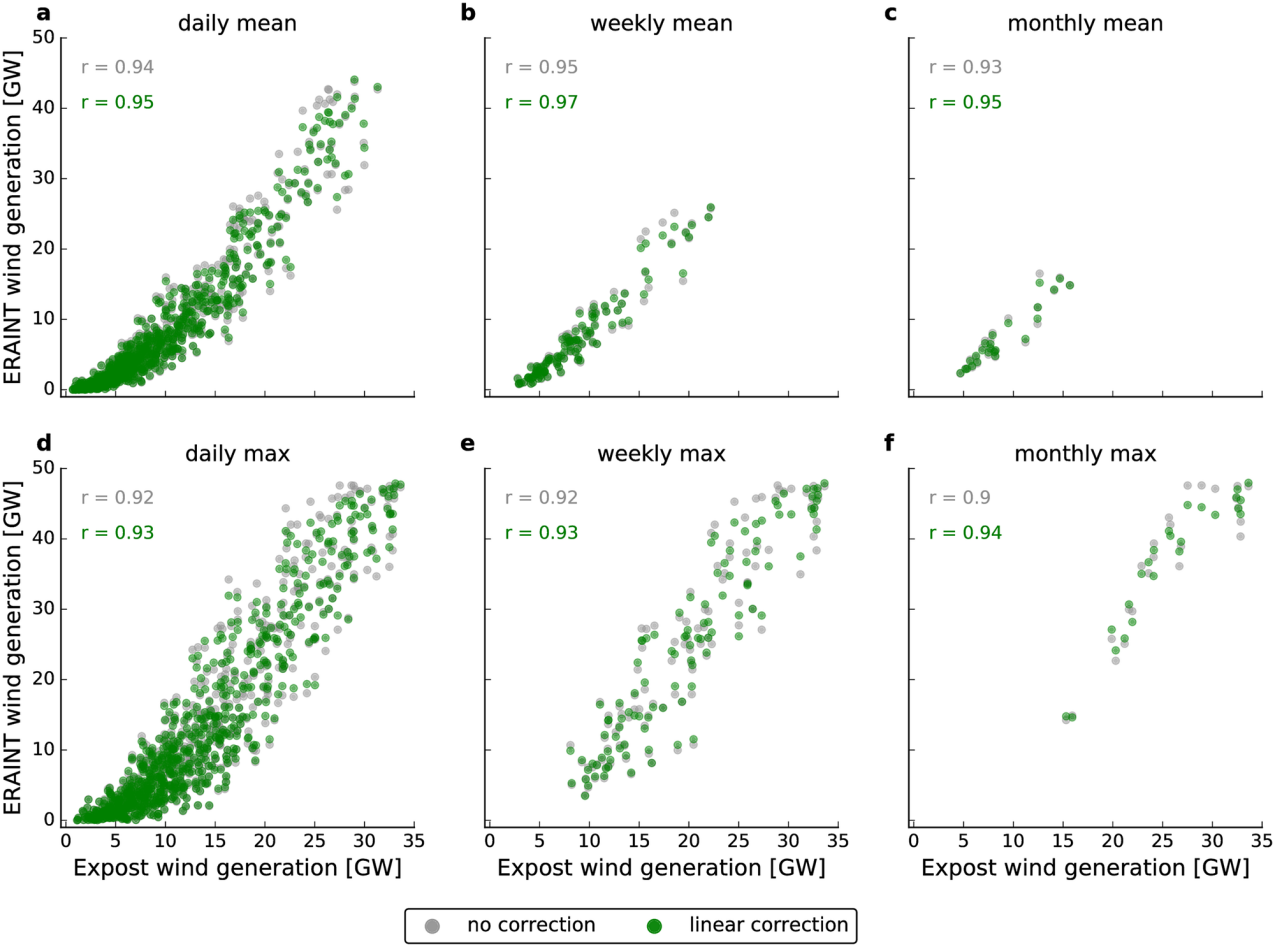
Scatter plots of ERAINT-based wind generation derived in this study versus expost wind generation as reported by German TSOs. Gray colors indicate ERAINT-based data that completely neglects capacity extension. Green denotes data that has been linearly adjusted for capacity increases. The Pearson correlation is given in the upper left area of each subplot. Columns represent different temporal aggregation levels ranging from daily (**a,d**) to weekly (**b,e**) and monthly (**c,f**) data. The upper line (**a-c**) shows mean values over the given interval while the lower line (**d-f**) represents maxima. All values are Germany-wide aggregates.

The ERAINT reanalysis provides near-surface wind speeds. In order to calculate wind generation from near-surface wind speeds, a couple of assumptions are made. These assumptions are later justified by comparison with measured wind generation data (see Fig 2). Following the approach described in [40], we first assume a power-law vertical wind profile with a fixed exponent ($v\left(z\right)=v\left(z=10\text{m}\right)\cdot {\left(\frac{z}{10\text{m}}\right)}^{\frac{1}{7}}$) and thereby neglect different stability regimes. Surface roughness is also neglected such that land cover and land-sea differences are not incorporated in the vertical scaling. They are, however, included in the derivation of the ERAINT dataset itself. Second, all wind turbines are assumed to be of the same kind and have a constant hub height of 80m. Third, wind park locations and sizes are assumed to be constant during the two-year period and taken as the end-2016 values from the Open Power System Database [53], see Fig 1. Keeping the installed wind capacities fixed allows us to isolate the effect of meteorological changes on wind power generation. Given that almost 10% new wind capacity was added in Germany during each of the last two years, the assumption of a steady state may seem crude. However, it is a well-accepted approach to assess non-stationary systems by studying steady-state cases first and include perturbations in time in a second step. Nevertheless, this approach can only be applied to relatively short periods of measured data. If the analysis was extended from a two-year measured time series to, for example, a ten-year series, the evolution of the wind parks became dominant and would have to be accounted for.

Again following [40], wind speeds at hub height are translated into wind generation using a simple power curve:

$$\begin{array}{c} P\left(v_{H}\right)=P_{0}\{\begin{array}{cc} 0, & \text{if}\;v_{H}<v_{i}\;\text{or}\;v_{H}>v_{0}\\ \frac{v_{H}^{3}-v_{I}^{3}}{v_{R}^{3}-v_{I}^{3}}, & \text{if}\;v_{I}\leq v_{H}<v_{R}\\ 1, & \text{if}\;v_{R}\leq v_{H}<v_{0} \end{array} \end{array}$$ (1)

where *v*_*H*_ denotes wind velocity at hub height and *v*_*I*_ = 3.5 m/s, *v*_*R*_ = 12 m/s, *v*_0_ = 25 m/s denote the cut-in, rated and cut-out velocity of the wind turbine, respectively. *P*_0_ is the installed capacity in the grid cell.

### Validation of generation timeseries

The validity of our approach is proven by comparison with measured data. In Fig 2, scatter plots of our wind generation time series from ERAINT versus ex post wind generation as reported by the four German TSOs are given. The ex post data was preprocessed by Open Power System Data and is freely available online [54]. It contains hourly German wind generation starting in summer 2009. However, we only use 2015 and 2016 here as these years are the focus of this investigation. We consider both different temporal sampling (from daily to monthly) and different sampling methods (mean or max values during the sampling period). Pearson correlations are always at least *r* = 0.9 and we hence conclude that our model captures the behavior of the real system sufficiently well. A linear correction of the ERAINT wind generation time series to include the effect of added capacities (given in green) further increases the correlations. For example, the correlation of daily means (Fig 2a) increases from *r* = 0.94 to *r* = 0.95. As correlations are already high without this correction, the remainder of this study is based on the uncorrected wind generation data. Furthermore, we observe a systematic deviation for small values of the ERAINT wind generation, where ex post data is higher. The direction of this mismatch can be explained by the spatial and temporal averaging in ERAINT: wind speeds within a 6-hour interval (or within a grid box) can well be above the cut-in velocity of the wind turbines even if the 6-hour (or grid box) average is lower. In fact, it is the very task of wind park planners to identify locations with above-average yields due to small scale effects (e.g. channeling or land-sea circulations). Wind turbines with a larger hub height and/or lower cut-in velocity further add to this mismatch. In contrast, in the realm of high generation, our approach yields higher values than the ex post analysis. Given that the ex post data accounts for curtailed generation that could not be fed into the grid (while our approach neglects curtailment), we expect such a tendency. In conclusion, we consider our approach well suited to capture system-wide effects and long-term developments while we also acknowledge the existence of systematic deviations of limited magnitude.

### Redispatch data

The redispatch time series is published by the German TSOs and it is available through a transparency platform [55]. They have hourly resolution and we utilize the 2015 and 2016 data. We refer to the redispatch timeseries as *R*(*t*). Although spatial information is included (such as the grid region that is affected or the plant that had to ramp up/down its generation), we consider the German aggregate only because we are interested in system-wide effects. In principle, redispatch can be subdivided into voltage-induced and current-induced redispatch. The latter is responsible for the majority of redispatch events. However, we found that our results are largely insensitive to restriction to current-induced redispatch and hence decided to evaluate all redispatch events. Moreover, the present analysis is based on redispatch *reduction* measures. In order to maintain the energy balance, a redispatch reduction measure requires ramping up plants elsewhere. Ramping up can be realized via redispatch increases or via reserve plants. As the latter strategy proved to be more efficient, the relative contribution of reserve plants increased during the period under investigation [56]. In focusing on reduction measures, we circumvent these regulatory changes. Redispatch events are associated with the point in time (day or week or month) when they were started.

### Receiver operating characteristics (ROC)

The scope of this paper is to analyze the dependency of redispatch and wind generation on different time scales using both standard correlation measures and a binary performance measure. We employ the binary measure because it seems plausible that there is a threshold-like behavior of the redispatch. If no wind park generates electricity, no wind-induced redispatch is expected. While wind speeds increase, parks ramp up their generation. As long as generation is small, no congestion in the grid occurs and hence there is still no redispatch. But once a certain level of wind power generation is exceeded, system stability would be affected and redispatch measures begin.

In order to test this hypothesis, we define a binary classifier *R*_pred_ as

$$\begin{array}{c} R_{\text{pred}}\left(t\right)=\{\begin{array}{c} 1\text{,}\;\text{if}\;G_{\text{Wind}}\left(t\right)\geq \sigma \\ 0\text{,}\;\text{if}\;G_{\text{Wind}}\left(t\right)<\sigma \end{array} \end{array}$$ (2)

where *G*_Wind_(*t*) is ERAINT-based wind generation at time *t* (see above) and *σ* ∈ [0, max(*G*_Wind_(*t*))] is a threshold value. Similarly, we define a binary redispatch time series *R*_bin_(*t*) as

$$\begin{array}{c} R_{\text{bin}}\left(t\right)=\{\begin{array}{c} 1\text{,}\;\text{if}\;R(t)\geq \theta \\ 0\text{,}\;\text{if}\;R(t)<\theta \end{array} \end{array}$$ (3)

where *θ* is another threshold value and *R*(*t*) denotes redispatch reduction at time *t* (see above). We choose *θ* such that a given percentage of *R*(*t*) is considered an event in *R*_bin_ (i.e. *R*_bin_ = 1). This formulation allows for an assessment of, for example, the 75th percentile (i.e. 25% strongest redispatch events).

We assess the capability of the model (Eq 2) to reconstruct the binary redispatch time series (Eq 3) using ROC analysis [57]. In a ROC curve, the true positive rate (TPR) is plotted against the false positive rate (FPR). TPR is defined as the number of correctly identified redispatch events (TP, true positives) divided by the number of redispatch events (P, positives):

$$\begin{array}{c} \text{TPR}=\frac{\text{TP}}{\text{P}}, \end{array}$$ (4)

where

$$\begin{array}{c} \text{TP}=\sum_{t}\{\begin{array}{c} 1\text{,}\;\text{if}\;R_{\text{pred}}\left(t\right)=1\;\text{and}\;R_{\text{bin}}\left(t\right)=1\\ 0\text{,}\;\text{otherwise} \end{array} \end{array}$$ (5)

and

$$\begin{array}{c} \text{P}=\sum_{t}R_{\text{bin}}\left(t\right). \end{array}$$ (6)

Similarly, FPR is defined as the number of erroneously predicted redispatch events (FP, false positives) divided by the number of non-redispatch events (N, negatives):

$$\begin{array}{c} \text{TPR}=\frac{\text{FP}}{\text{N}}, \end{array}$$ (7)

where

$$\begin{array}{c} \text{FP}=\sum_{t}\{\begin{array}{c} 1\text{,}\;\text{if}\;R_{\text{pred}}\left(t\right)=1\;\text{and}\;R_{\text{bin}}\left(t\right)=0\\ 0\text{,}\;\text{otherwise} \end{array} \end{array}$$ (8)

and

$$\begin{array}{c} \text{N}=\sum_{t}\{\begin{array}{c} 1\text{,}\;\text{if}\;R_{\text{bin}}\left(t\right)=0\\ 0\text{,}\;\text{otherwise} \end{array}. \end{array}$$ (9)

A random classifier would create values along the diagonal, whereas a perfect classifier is given by a true positive rate of 1 and a false positive rate of 0. As a scalar performance measure, we calculate the area under the curve (AUC) which can be identified with ‘the possibility that the classifier will rank a randomly chosen positive instance higher than a randomly chosen negative instance’ [57]. The question we ask is: Given a certain redispatch threshold *θ*, how much of the binary redispatch time series can be explained based on the wind time series? Note that *θ* is predefined by us, while different values of *σ* are used to construct the ROC curve.

### Circulation weather types (CWTs)

This approach allows us to relate our findings to large-scale meteorologic conditions. In order to study the connection of redispatch variability and wind generation variability, we investigate the dependency of redispatch on different typical pressure regimes over Germany. The approach is based on a CWT classification [58] of the ERAINT dataset. The classification is centered over Germany, is representative for Central Europe and comes with a daily temporal resolution. The method separates eight directional CWTs (North:N, Northeast:NE, East:E etc.) and four non-directional ones (Cyclonic:C, Mixed Cyclonic:Mixed C, Anticyclonic:AC, Mixed Anticyclonic:Mixed AC). Further explanations can be found in [59]. The methodology has repeatedly been applied for energy-related purposes [31, 40, 60, 61].

## Results

### Natural variability of wind generation

**Fig 3:**
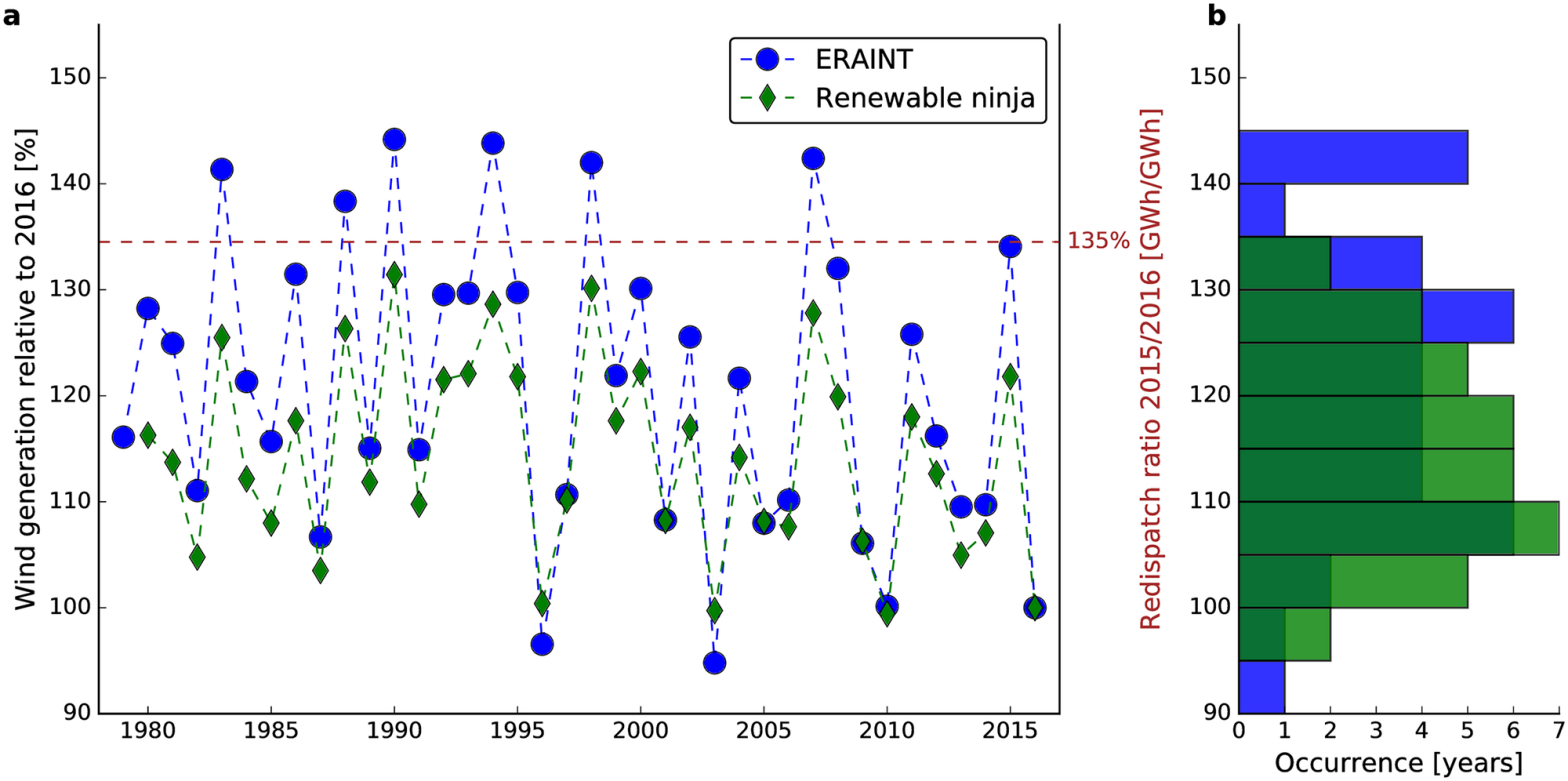
Natural variability of wind generation relative to 2016 in Germany. Time series of wind generation (**a**) and its distribution (**b**). Wind park configuration is kept constant throughout the entire timespan such that variations are solely rooted in wind variability. Blue denotes our own calculations and green indicates the renewables.ninja dataset [45]. The dashed brown line shows the ratio of 2015 to 2016 redispatch volume.

Fig 3 shows the time series of annual wind generation based on ERAINT. For validation purposes, it also includes version 1.1 wind generation data from renewables.ninja [45]. The renewables.ninja dataset starts in 1980 such that 1979 is only covered in our calculations. In Fig 3, renewables.ninja data is normalized such that its 2016 value coincides exactly with the ERAINT-based 2016 relative wind generation. There is quasi-perfect agreement between both time series in terms of the direction of changes between years. However, the magnitude of interannual changes in renewables.ninja is generally smaller. This may be due to differences in resolution. The underlying MERRA-2 reanalysis [62] has a fivefold coarser resolution than the ERAINT output used here, resulting in 25 ERAINT grid boxes per MERRA-2 grid box and thus a less realistic representation of spatial variability. This effect is, however, weakened or compensated for since [45] interpolated wind speeds to the wind park locations. However, the MERRA-2 reanalysis has a sixfold higher temporal resolution (hourly) such that a more realistic representation of fast changes is expected. Other differences in the approaches include that [45] accounted for different turbine types, interpolated vertically by fitting a logarithmic wind profile, applied a bias correction and used the wind park configuration of 2015.

Based on a detailed representation of the end-2015 wind parks, [50] followed a different approach to handle the coarse resolution of the MERRA reanalysis in deriving the EMHIRES dataset. They applied statistical downscaling over land to account for small-scale effects like complex topography and reported enhanced agreement with measured data. Over the ocean, no downscaling was applied because ocean surface conditions are sufficiently homogeneous.

The energy generated from the end-2016 German wind fleet fluctuates strongly in time (see Fig 3). In comparison to the weakest years, an additional 40% wind energy can be generated in the strongest years (30% based on renewables.ninja). In particular, there was substantially less wind generation in 2016 compared to 2015. This agrees perfectly with elevated redispatch costs in 2015: the ratio of 2015 to 2016 redispatch volume is given as a dashed brown line and fits extremely well with the ratio of 2015 to 2016 wind generation. Although the closeness of the agreement is likely a coincidence, it shows that both the direction of change and magnitude of ERAINT-based wind generation and measured redispatch volumes are in agreement.

Moreover, it is evident that 2016 was at the lower edge of all years covered in both datasets. If no regulatory changes were made to the system, an increase in redispatch would hence be very likely in 2017 (yet not guaranteed) and the TSO TenneT has already reported an increase of costs in spring 2017 [63]. Based on the historical record, the increase could even be higher than the drop from 2015 to 2016. However, this obviously depends on the actual characteristics of 2017 wind fields which we do not claim to forecast here.

### Extent to which redispatch can be traced back to wind generation

In the following subsections, we quantify the dependency between redispatch energy and wind generation based on correlations and receiver operator characteristics. Furthermore, we investigate the underlying meteorological variability by means of circulation weather types.

#### Correlation analysis

**Fig 4:**
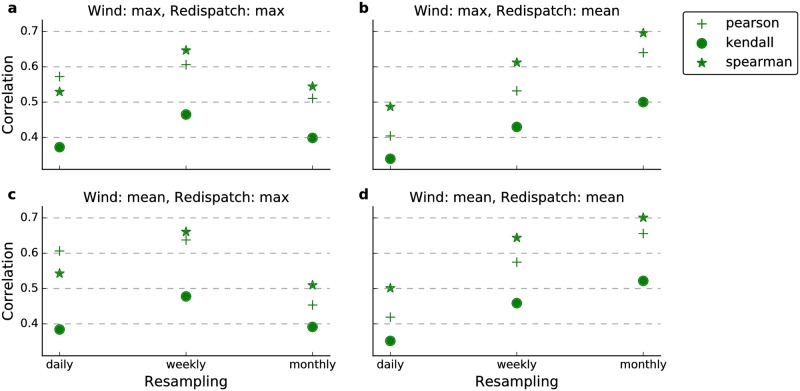
Correlation measures between wind generation and redispatch timeseries. Different panels show different temporal sampling methods. The upper line (**a,b**) uses maximum values of the wind generation time series, while the lower one (**c,d**) uses the mean. The left column (**a,c**) employs maximum values for redispatch resampling, while the right (**b,d**) is based on the mean. Markers denote the correlation measure employed (Kendall’s *τ*, Spearman’s *ρ* or the Pearson correlation coefficient *r*). Horizontal dashed lines are given for ease of interpretation.

We compare the wind generation and redispatch time series by evaluating three different correlation measures in Fig 4. They are the linear Pearson correlation, the non-linear Spearman’s rank and Kendall’s rank correlation (e.g., [64]). We assess different levels of temporal aggregation from daily to monthly. Within the resampling window, we either use the mean or the maximum value for both time series. This yields four possible combinations of averaging procedures as presented in the subplots (Fig 4a–4d).

Generally, we report moderate to strong positive correlations. This statement holds for the linear Pearson measure as well as for the non-linear Spearman’s and Kendall’s rank correlations. The mean redispatch volume follows wind generation better if averaged over long periods (i.e. weeks or months) and reaches values of around 0.7 (Fig 4b and 4d). This tendency is in agreement with the results of the receiver operating characteristics (cf. below). In contrast, model performance for the redispatch maxima deteriorates on a monthly level (Fig 4a and 4c) indicating that the maximum redispatch events within a month are not strongly connected to the monthly wind generation. However, we want to highlight the monotonous increase in correlations for the mean redispatch and mean wind (Fig 4d) because it shows that mean wind generation can be translated into mean redispatch. Due to this monotony, we expect the seasonal and annual values to be even higher than the monthly values. We are thus confident that average wind generation has good predictive skill for average redispatch on a seasonal and annual basis.

#### Receiver operating characteristic (ROC) analysis

**Fig 5:**
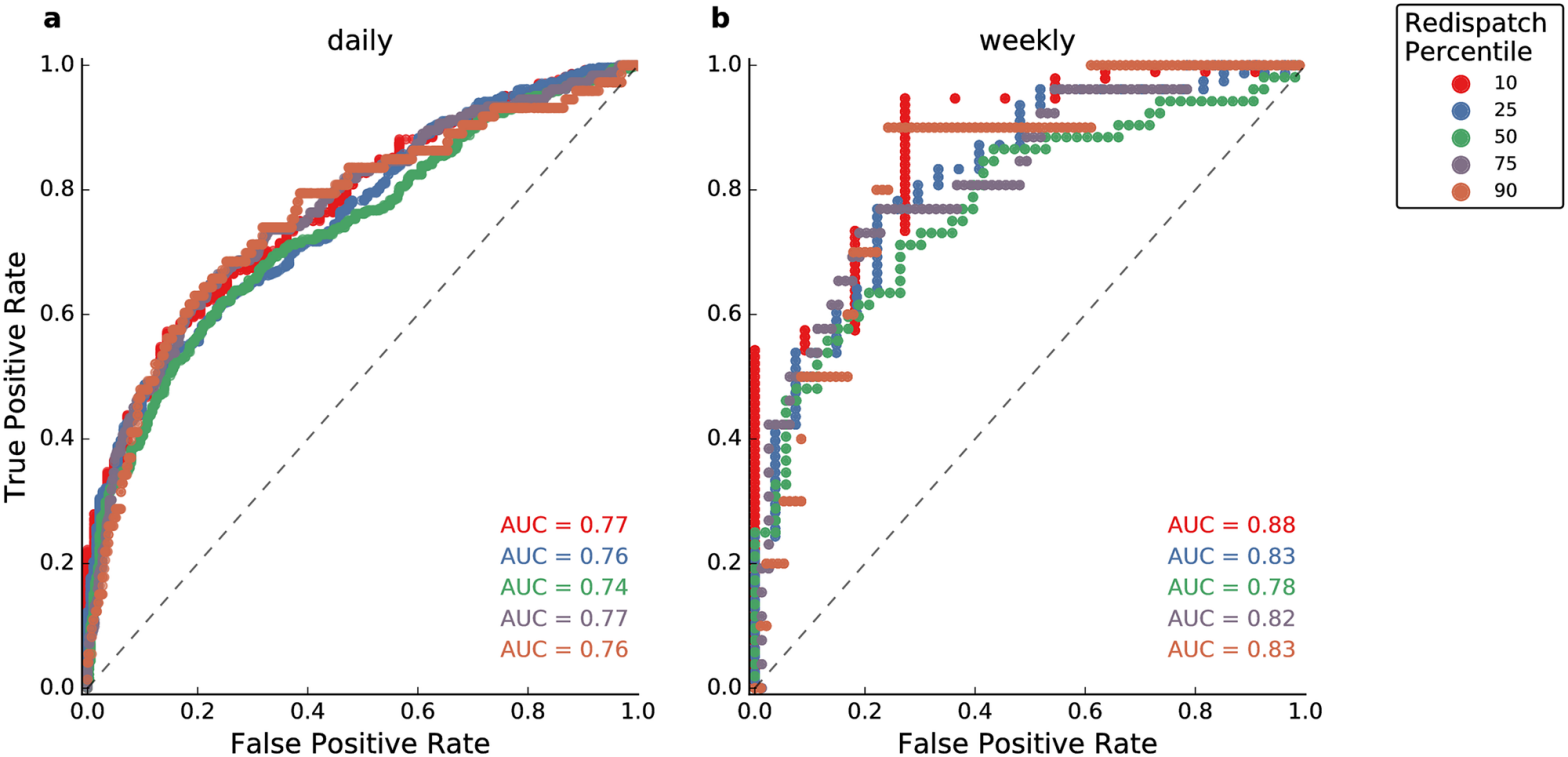
Receiver operator characteristic curve testing the performance of wind generation as a binary classifier for redispatch. Both wind and redispatch timeseries were resampled based on daily means (**a**) and weekly means (**b**).

Based on a ROC analysis (see Methods and data), we report that the wind generation time series can partly explain redispatch as shown in Fig 5. Both the redispatch and the wind generation time series were resampled using the mean over a time interval (day or weak) here. The analysis reveals that the classifier performs reasonably for daily values (Fig 5a). Showing AUC of 0.75 ± 0.02, it is distinctly better than a random classification (AUC = 0.5) and hence there is clearly a signal of the wind generation in the daily series. On a weekly basis, the model performance is distinctly better (Fig 5b). This could be indicative of redispatch measures being scattered around the meteorological events causing them. Sometimes a redispatch is scheduled prior to the strong wind event, sometimes it lags behind. This scattering might be caused by uncertainties regarding the timing of strong wind events and it could also be affected by inertia of the conventional power system (such as long ramping times which require system operators to act well ahead of the actual event). While a mis-association of events on a daily basis follows, the coarser consideration based on weeks weakens this effect.

On the weekly basis, the classifier performs very well in separating the 10th percentile (AUC = 0.88). This means that we can isolate low redispatch weeks well. Very high redispatch weeks can also be separated well (AUC = 0.83 for the 90th percentile and AUC = 0.82 for the 75th percentile). Seperating the 50th percentile, however, is not quite as reliable (AUC = 0.78).

The ROC analysis was also performed for resampling methods other than the mean (see S1 File). The results are mostly insensitive to changes in the sampling method with one interesting exception: the mean and max wind time series are skillful in determining the 90th percentile of max redispatch events on a daily basis (AUC = 0.89 and AUC = 0.88). In other words, the highest single redispatch event on a daily level can be attributed well to high wind generation, independent of the resampling method of the wind time series.

#### Variability of weather patterns

In the two preceeding subsections, we showed that redispatch is related to the natural variability of near-surface wind conditions. Wind patterns over Europe in turn are associated with large-scale weather types. We therefore investigate the dependency of redispatch on CWTs (see Methods and data) by calculating the relative contributions of individual CWTs to overall redispatch.

**Fig 6:**
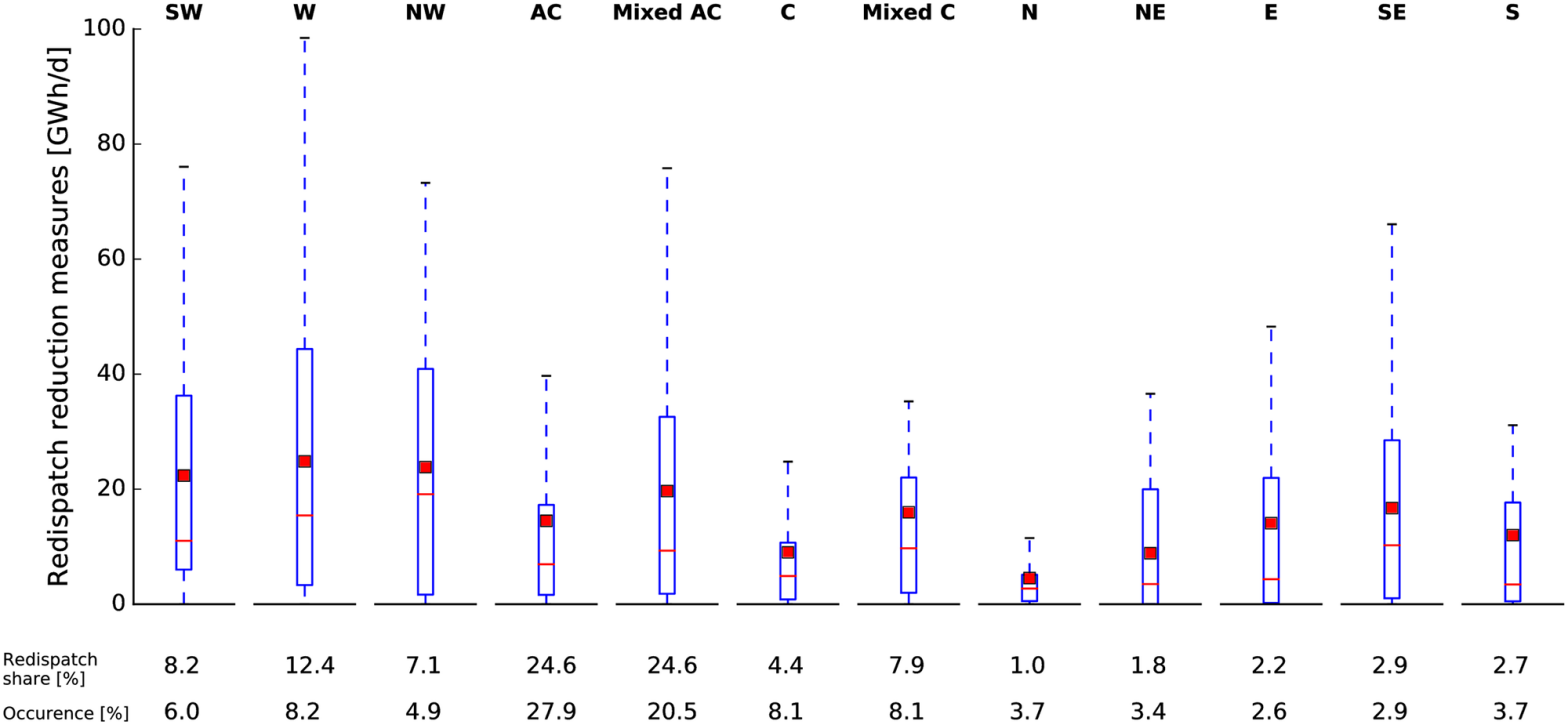
Daily redispatch decomposition for different CWTs. Each boxplot shows the statistics of 2015 to 2016 daily redispatch data differentiating between the CWT prevalent on the respective day. Blue boxes indicate the 25th to 75th percentile and error bars indicate the 5th to 95th percentile. A red thin line denotes the median while the mean is given as a red thick line. Below the plot, the share of redispatch and the relative occurence of each CWT are given. Abbreviations denote the different CWTs. In addition to the directional CWTs (e.g. southwest, SW), there are anticyclonic (AC) and cyclonic (C) CWTs and also mixed versions of them.

CWTs of type southwest (SW), west (W) and northwest (NW) are characterized by high levels of redispatch (see Fig 6) and we refer to them collectively as westerly CWTs. 27.7% of redispatch happened during such westerly CWTs although they only occurred during 19.1% of the time. From a meteorological point of view, this finding is plausible since westerly CWTs are typically accompanied by relatively large pressure gradients and strong winds. The largest contributors to redispatch in absolute terms are anticyclonic (AC, 24.6%) and mixed anticyclonic (Mixed AC, 24.6%) configurations but they also occur most often (27.9% and 20.5%, respectively). Therefore, they are less redispatch-intense than westerly CWTs. Furthermore, the 95th percentile is highest for the western (W) CWT, indicating that the strongest redispatch events occur during this CWT. Weak redispatch is to be expected under CWTs of type cyclonic (C), north (N), northeast (NE), east (E) and south (S). Interestingly, all distributions of redispatch reduction measures given a certain CWT have positive skewness since the average is always greater than the median.

**Fig 7:**
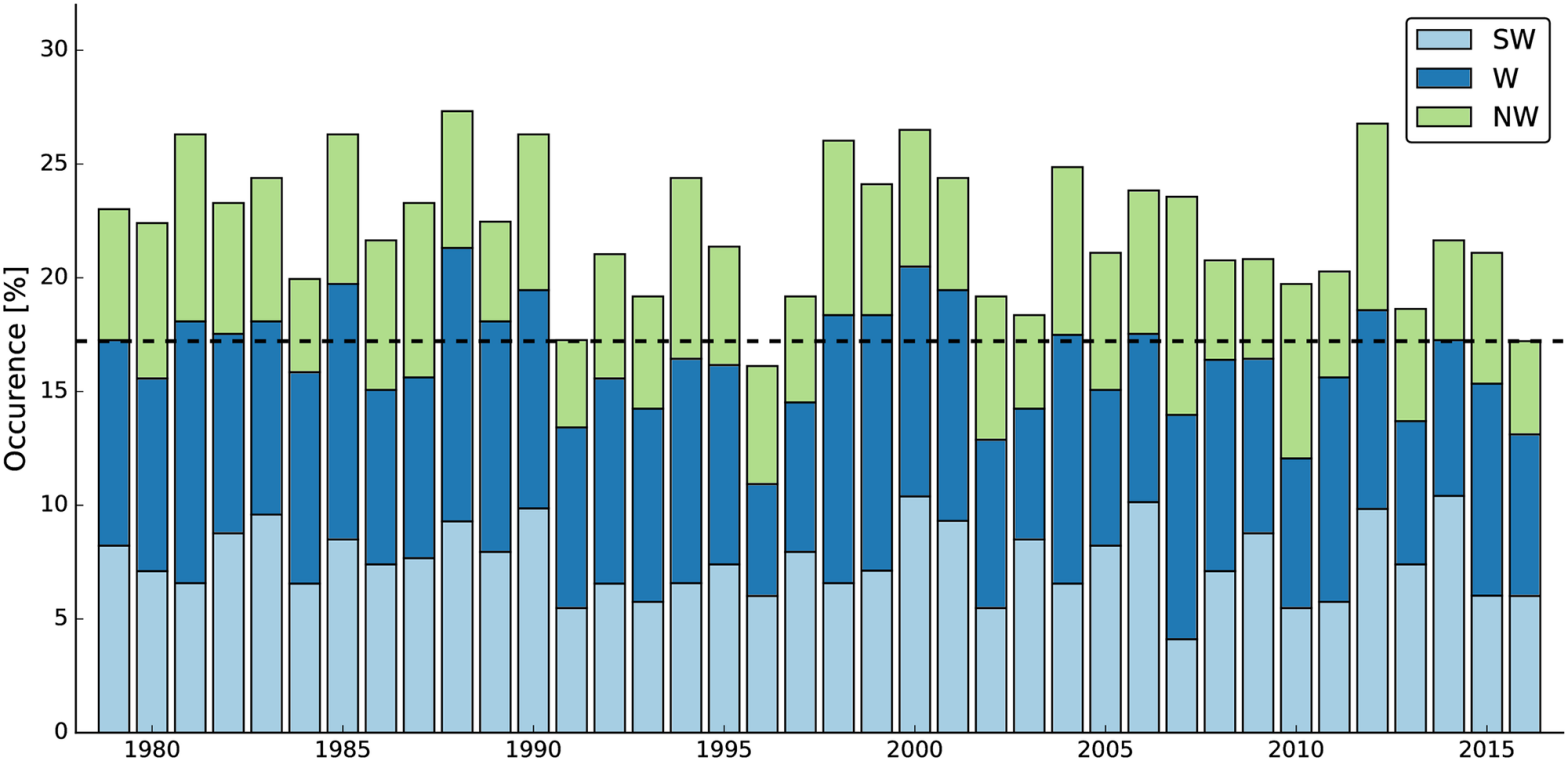
Variability of westerly CWTs. The occurrence is given in percent and is based on a daily CWT classification. The horizontal dashed line indicates the 2016 value and is plotted for convenience.

Having found that westerly CWTs are characterized by elevated levels of redispatch, an assessment of their variability is insightful. Fig 7 shows that westerly CWTs prevail between 16% and 27% of the year in ERAINT, indicating a similar range of variability as for wind generation (cf. Fig 3). With respect to the last two years, it is clearly visible that 2015 had more westerly CWTs than 2016 in line with the downward shift of redispatch volume. Moreover, 2016 is among the lowest years on record in terms of westerly CWT occurrence. Only 1996 lies below and 1991 shows an almost identical value. The remaining 34 years on the record are characterized by higher values. Hence, 2016 has an exceptionally low occurrence of westerly CWTs.

## Discussion

As outlined in the Results section, we assessed the variability of annual wind energy generation due to natural climatic variability. We found annual variability to be substantial and argue that it is an important characteristic of power systems with a high share of wind generation, in agreement with the literature (e.g., [65–67]). Capturing this variability does not necessarily introduce the need to use extensive time series of volatile renewable generation directly. Instead, a high level of the fluctuations can be reproduced by representative days based on a hierarchical clustering algorithm [68]. Representative days can reduce the computational costs substantially, although the required number of representative days depends on the question to be answered. [69] argued that benefits from more realistic time resolutions dominate over benefits from the inclusion of techno-economic details. They thus advocate that model developers should aim at improving the temporal resolution.

In this context, it appears problematic that some state-of-the-art integrated assessment models [70, 71] and policy-relevant studies on a national level (e.g., [72]) still use single or representative weather years as input for their calculations. By ignoring interannual generation variability in the analysis, results can be biased. For example, Elsner et al. [72] use 2008 weather data although this particular year was above average in terms of mean wind generation (see Fig 3).

Moreover, in a climate system away from equilibrium, interannual wind variability may well change in the future. [31] showed that the climate change impact on interannual variability is subject to large inter-model spread in a large climate model ensemble (CMIP 5) and is hence substantially uncertain. However, the increase of inter-annual wind variability over Germany in downscaled climate projections can be as high as 30% under strong climate change at the end of the 21st century [73]. In a study aiming to establish a framework for economic assessments of climate change impacts on electricity generation, [74] also covered potential changes of inter-annual variability.

Supporting our results, a previous, non-peer-reviewed study, also found wind generation and redispatch to be substantially correlated [75]. Based on daily ex post data, they reported a Pearson correlation coefficient of 0.65 in the period April 2013 to March 2017. In comparison to our values, their correlation coefficient is generally higher, although we obtain comparable Pearson correlations for some sampling methods (see Fig 4a and 4c). This slight discrepancy is not surprising as the considered time intervals differ.

The relatively high correlations motivate a linear model of the redispatch

$$\begin{array}{c} R\left(t\right)=a\cdot G_{\text{Wind}}\left(t\right)+b, \end{array}$$ (10)

where *R*(*t*) is redispatch at time *t*, *G*_Wind_(*t*) is wind generation, *a* and *b* are constants. Similar to the correlations themselves, the skill will increase with coarser resampling and we expect good skill at monthly or even annual resampling.

In addition, Fig 3 shows that a 35% increase in annual wind generation translates into a 35% increase in the annual redispatch energy from 2016 to 2015:

$$\begin{array}{c} \frac{R(2015)}{R(2016)}=\frac{G_{\text{Wind}}\left(2015\right)}{G_{\text{Wind}}\left(2016\right)}. \end{array}$$ (11)

A combination of Eqs 10 and 11 yields *b* = 0. The ratio of redispatch between two weather years *y*_1_ and *y*_2_ is hence identical to the ratio of wind generation

$$\begin{array}{c} \frac{R(y_{1})}{R(y_{2})}=\frac{a\cdot G_{\text{Wind}}\left(y_{1}\right)+b}{a\cdot G_{\text{Wind}}\left(y_{2}\right)+b}=\frac{a\cdot G_{\text{Wind}}\left(y_{1}\right)}{a\cdot G_{\text{Wind}}\left(y_{2}\right)}=\frac{G_{\text{Wind}}\left(y_{1}\right)}{G_{\text{Wind}}\left(y_{2}\right)}. \end{array}$$ (12)

As a consequence, the range of variability of wind generation is identical to the range of redispatch variability. Fig 3 thus allows the latter to be quantified as between 95% and 145% of the 2016 values for the entire period covered by ERAINT. Admittedly, a stringent test of this statement would require freezing the electricity system as of today and studying its evolution in different weather years forever. This is obviously not feasible, leaving us with incomplete knowledge and leading to a standard verification dilemma of numerical models in the earth sciences [76]. Despite this and in line with [76], we argue that our finding provides a useful heuristic. It is furthermore obvious that this linear model is no longer valid after substantial changes are made to the current system, for example via transmission line extension or modifications of the guiding principle of the dispatch.

The CWT analysis revealed that redispatch is particularly high during westerly flows in line with meteorological intuition. In terms of planning, this finding could be employed beneficially for the overall system performance. For example, the addition of wind parks that are optimized to yield maximum output under non-westerly CWTs will have a substantially smaller effect on redispatch energy. However, the challenge here lies in the identification of suitable locations which still have sufficiently high capacity factors to prove economically viable.

It is furthermore interesting to note that the stochasticity of the wind generation signal can have a large influence on public perception. For example, the high redispatch costs in 2015 led to extensive media coverage across the entire spectrum from tabloids [77], online-only [78] and weekly magazines [23] to standard newspapers [22, 79, 80]. The language in the articles is heated, for example, it is stressed repeatedly that wind park operators are paid for idleness [24, 25] and the word ‘battle’ (‘Kampf’) is used in some headlines [78, 80]. During this public debate, the German minister for economics and energy, Sigmar Gabriel, is cited expecting a further 50% increase in overall grid-management costs to € 1.5 billion in 2016 [79]. In light of the lively public debate with respect to costs, it seems plausible that the strong wind year 2015 had an impact on policy making. In particular, it is questionable whether the 2016 EEG reform [27] would have been the same, had 2015 been a rather calm wind year.

## Conclusion and policy implications

The German power system is undergoing a fast and drastic transition towards renewables. As an unpleasant side-effect, redispatch measures which aim at securing stability of the power grid have been used more extensively and reached an annual cost of around € 400 million in 2015. The subsequent year was characterized by a sharp decline of these costs. We report that much of this decline is rooted in natural climatic phenomena and is hence stochastic. Our confidence in this finding is very high since our argumentation is based on multiple lines of evidence.

First, 2015 was a strong wind year in terms of annual wind power generation and 2016 was a weak one compared to a 37-year reanalysis time series. Second, ROC analysis suggests that mean wind generations are a suitable classifier to determine redispatch on long time scales (i.e. weeks and beyond). On these time scales there is even a considerable linear and rank correlation between the wind generation time series and the redispatch time series. Hence, a weak wind year translates into a low redispatch year. Third, redispatch is found to be high during westerly CWTs and those were more abundant in 2015 than in 2016.

Over the 37 years covered by the ERAINT dataset, we found annual wind generation variability ranging from 95% to 145% of the 2016 values. Following a simple linear model calibrated by the 2015 and 2016 redispatch energy, this also implies variability of redispatch energy in the same range.

It should be noted that all these conclusions are bound to the current power system. This is true both in terms of physical constraints and management aspects. While the physical constraints, such as transmission limits or locations of generators, are hard facts (i.e. evolve on long time scales of multiple years to decades), system management is, amongst others, subject to laws, regulations and economic incentives. Given political will, the latter can be adapted faster than the physical system. For example, including limited transmission capacities in deriving the dispatch would clearly be a game changer and may have the potential to reduce redispatch dramatically. This is because the current guiding principle of the dispatch is based on the assumption that its outcome is mostly compatible with transmission grid constraints. Conflicts with these constraints are assumed to be minor. If they occur, the redispatch will ensure system stability. However, given the continued addition of renewables, and the relatively slow pace of transmission line extensions, this assumption is challenged as congestion becomes more important. As a consequence, the minimization of power generation costs does not necessarily coincide with optimum operation of the power system. Instead of solving dispatch problems via the redispatch, it thus may be favorable to include the physical constraints into the dispatch as in optimal power flow algorithms (e.g., [81]). More generally, reducing the time window of the dispatch may have a positive effect due to less uncertain forecasts. Also, efficient carbon pricing may make gas plants economically superior to coal plants and hence decrease average ramping times in the dispatch.

In order to understand redispatch more precisely, a possible next step would be to resolve the national grid explicitly. This would allow congestion owing to renewable generation to be simulated and subsequently compared to the redispatch energy reported by TSOs. It would also be interesting to expand the assessment to the time series of the feed-in management and the grid reserve. Moreover, the role of electricity exports could be assessed. As hypothesized earlier, redispatch could be exacerbated by exports that introduce additional loads to the electricity grid in times of high renewable generation.

Independent of the exact design of the future power system, variability will be a fundamental property of it. This is true for any power system based on renewables and by no means limited to the German example studied here. Therefore, we suggest a stronger consideration of uncertainty and natural variability in any assessment of the current energy system. As public perception can be affected strongly by single events like the 2015 peak in redispatch costs, short-sighted reactions might follow. They are to be avoided because hectic weakening of renewable expansion in times of high redispatch years and strengthening of renewable expansion during low redispatch years may substantially harm the energy transition. This is because renewable energy companies need stable ground to build on [82]. There is hence a requirement for robust decision making [83] incorporating interannual variability of the wind resources. In order for science to be helpful in this, research should aim at better understanding and quantifying climate-induced variability on different time scales including years and decades. Additionally, closer collaboration between energy and climate modelers is urgently needed.

## Supporting information

S1 File ROC analyses under different resampling methods. (PDF)

S2 File Wind generation time series. (CSV)
