# Supplementary material for: Natural wind variability triggered drop in German redispatch volume and costs from 2015 to 2016
Source: PLoS One. 2018 Jan 12;13(1):e0190707. doi: 10.1371/journal.pone.0190707 (PMC5766128; doi:10.1371/journal.pone.0190707)
Supplement: S1 File — (PDF) [file pone.0190707.s001.pdf]

S1: ROC analyses under different resampling methods

Supporting information to "Natural wind variability triggered drop in German redispatch volume and costs from 2015 to 2016" by Wohland et al.

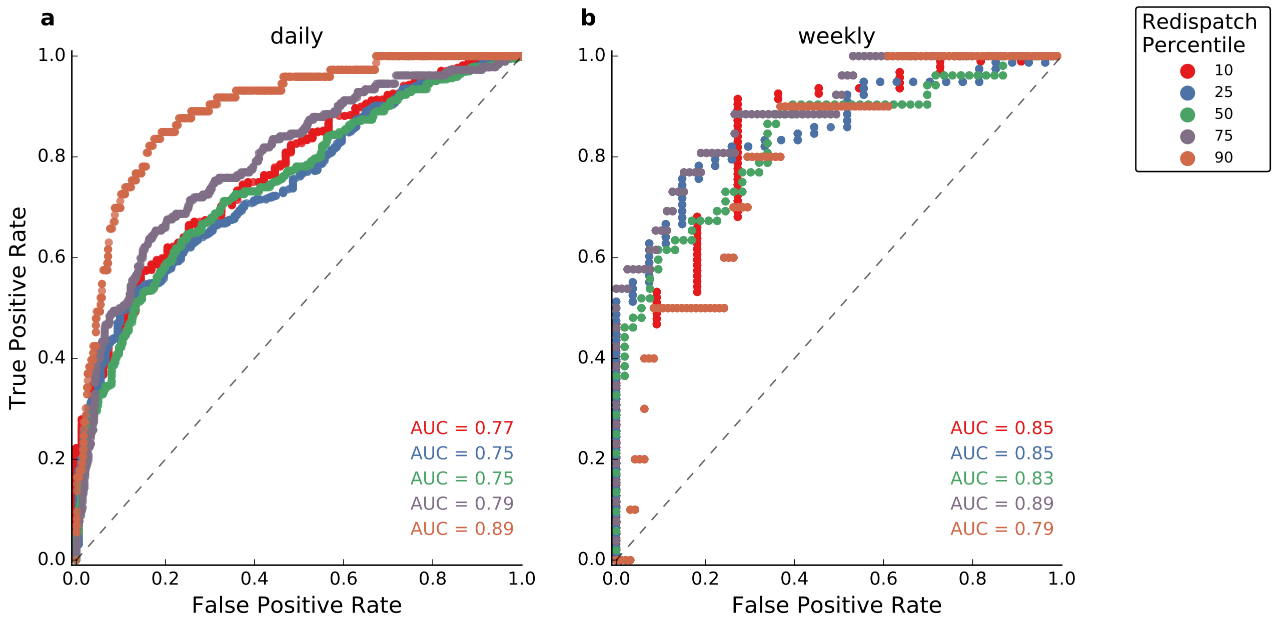

Figure S1: **Same as Fig. 5 in the manuscript but for mean resampling of wind and max resampling of redispatch.**

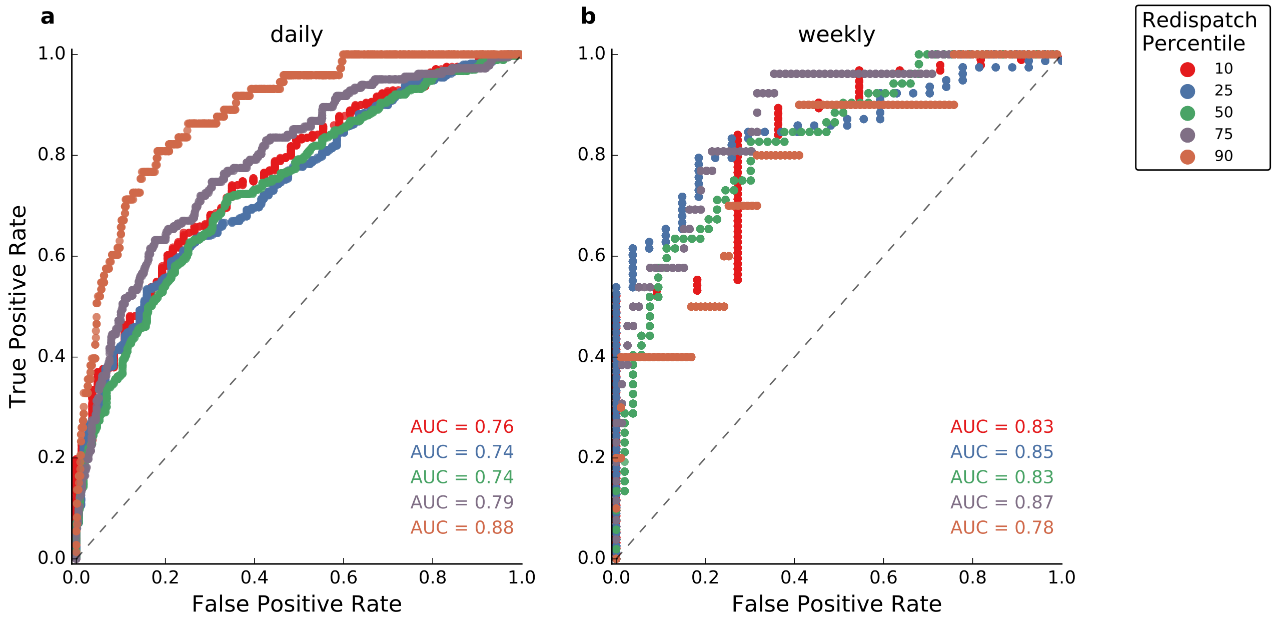

Figure S2: **Same as Fig. 5 in the manuscript but for max resampling of wind and max resampling of redispatch.**

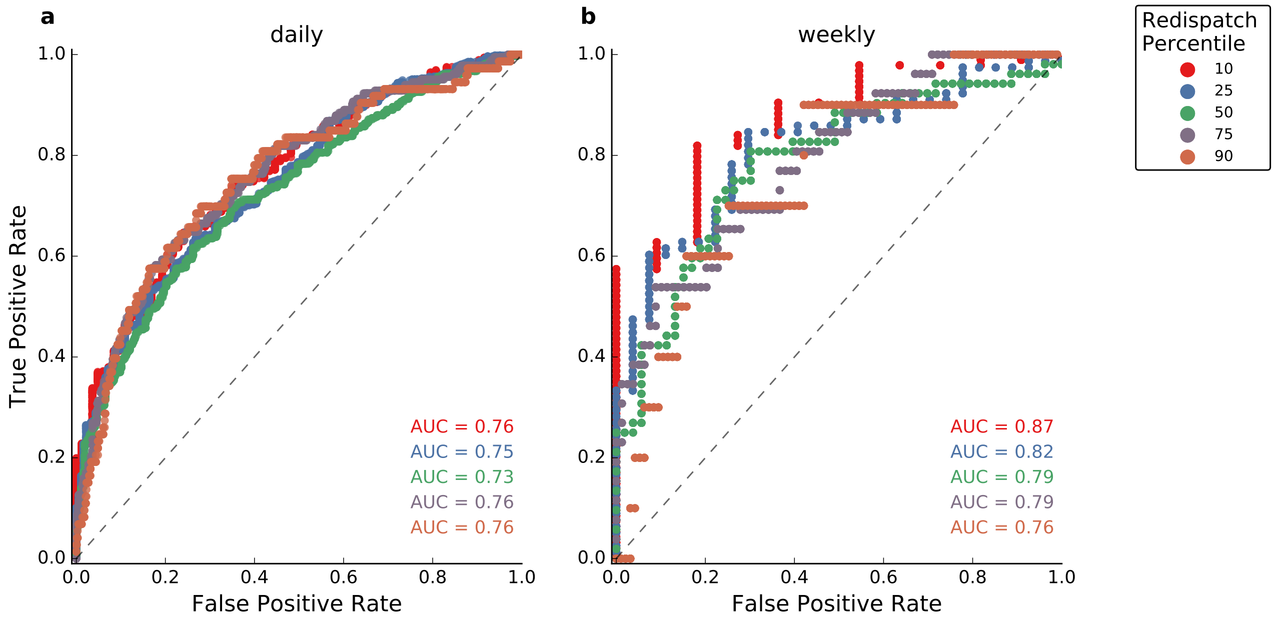

Figure S3: **Same as Fig. 5 in the manuscript but for max resampling of wind and mean resampling of redispatch.**
